# The *qacC* Gene Has Recently Spread between Rolling Circle Plasmids of *Staphylococcus*, Indicative of a Novel Gene Transfer Mechanism

**DOI:** 10.3389/fmicb.2016.01528

**Published:** 2016-09-27

**Authors:** Trudy M. Wassenaar, David W. Ussery, Hanne Ingmer

**Affiliations:** ^1^Molecular Microbiology and Genomics ConsultantsZotzenheim, Germany; ^2^Department of Biomedical Informatics, University of Arkansas for Medical SciencesLittle Rock, AR, USA; ^3^Department of Veterinary Disease Biology, Faculty of Health and Medical Sciences, University of CopenhagenCopenhagen, Denmark

**Keywords:** gene mobility, *qac*, SMR, *S. aureus*, evolution, DNA transfer, rolling circle plasmids

## Abstract

Resistance of *Staphylococcus* species to quaternary ammonium compounds, frequently used as disinfectants and biocides, can be attributed to *qac* genes. Most *qac* gene products belong to the Small Multidrug Resistant (SMR) protein family, and are often encoded by rolling-circle (RC) replicating plasmids. Four classes of SMR-type *qac* gene families have been described in *Staphylococcus* species: *qacC, qacG, qacJ*, and *qacH*. Within their class, these genes are highly conserved, but *qacC* genes are extremely conserved, although they are found in variable plasmid backgrounds. The lower degree of sequence identity of these plasmids compared to the strict nucleotide conservation of their *qacC* means that this gene has recently spread. In the absence of insertion sequences or other genetic elements explaining the mobility, we sought for an explanation of mobilization by sequence comparison. Publically available sequences of *qac* genes, their flanking genes and the replication gene that is invariably present in RC-plasmids were compared to reconstruct the evolutionary history of these plasmids and to explain the recent spread of *qacC*. Here we propose a new model that explains how *qacC* is mobilized and transferred to acceptor RC-plasmids without assistance of other genes, by means of its location in between the Double Strand replication Origin (DSO) and the Single-Strand replication Origin (SSO). The proposed mobilization model of this DSO-*qacC*-SSO element represents a novel mechanism of gene mobilization in RC-plasmids, which has also been employed by other genes, such as *lnuA* (conferring lincomycin resistance). The proposed gene mobility has aided to the wide spread of clinically relevant resistance genes in *Staphylococcus* populations.

## Introduction

During investigations of *qacC*, a gene that provides resistance of *Staphylococcus aureus* to quaternary ammonium compounds (qac) often used as biocides or disinfectants, we noticed an unusual pattern of sequence conservation amongst various isolates. QacC and related proteins QacG, QacH, and QacJ belong to the protein family of Small MultiResistant proteins (SMR) (Lyon and Skurray, 1987; Wassenaar et al., 2015); they are typically around 100 amino acids long and contain two trans-membrane domains. It is assumed they form dimers in the membrane to create a pore through which their substrates are removed from the cell. Some SMR-members of the Qac family are found on long (>30,000 bp) conjugative plasmids, but most are present on small (< 4000 bp) plasmids that are maintained by rolling-circle replication. Rolling circle (RC-) plasmids start their replication at a nick-site of a so-called DSO (double-strand replication origin), producing the plus, or leading strand, after which the minus strand is produced from a separate SSO locus (Khan, 2005) (also known as *palA* element, Gruss et al., 1987). RC-plasmids of *Staphylococcus* species typically contain only the gene required for initiation of replication, but sometimes a second gene (like *qacC*) is present, and occasionally longer RC-plasmids contain multiple genes.

Studies on the evolutionary relationship of the QacC locus with different plasmids of *Staphylococcus* spp. date back more than a decade and the mode of spread of this gene remains unexplained. That *qacC* could be transferred between plasmids had been observed as early as 1995, when it was proposed that the *qacC* locus of conjugative plasmid pSK41 was the prototype of a so-called *qacC* cassette (Leelaporn et al., 1995). This view was adopted by others, with the *qacC* locus of pKS41, which contains a direct repeat flanking *qacC*, designated as “Type 1” and loci with different flanking regions surrounding *qacC* classified as “Type 2” and “Type 3” (Bjorland et al., 2001; Alam et al., 2003). The nomenclature was based on a partial sequence of pSK41 that unfortunately had been wrongly assembled. The availability of the complete sequence of pSK41 and closely related plasmids, as well as a large number of sequenced *qacC* and other *qac* RC-plasmids, enabled a new comparative analysis. Since most sequence records are available for *qacC*, this gene was the main focus of the comparison presented here. The nomenclature originally introduced for *qacC*-bearing plasmids can no longer be maintained. Based on the observed homology patterns amongst different plasmids, we propose a novel mechanism for gene mobilization, which is not exclusive to *qacC*, as a second resistance gene was identified that most likely also spread using the proposed mechanism.

## Methods

All plasmid sequences used in this study were downloaded from GenBank. Sequence similarity searches were performed by BlastP and BlastN at NCBI. Multiple alignments were produced by Clustal W or Muscle. Circular homology plots were based on sequence alignments with gaps >12 nucleotides removed before calculating the percentage homology using a window of 30 nucleotides. DNA structural properties were assessed as previously described in Pedersen et al. (2000).

## Results

### QacC plasmid types i and ii

All recorded QacC proteins encoded by RC-plasmids are strictly conserved. Seventy GenBank entries describe identical proteins, sequenced from different staphylococcal plasmids. Only two amino acid (aa) substitutions have thus far been described: an A → S substitution at aa position 9 has been recorded five times (four from *Staphylococcus warneri* plasmids and one from a non-speciated *Staphylococcus* spp.) and a change of I → T at position 30 has been found twice in *S. epidermidis* plasmids.

**Figure 1:**
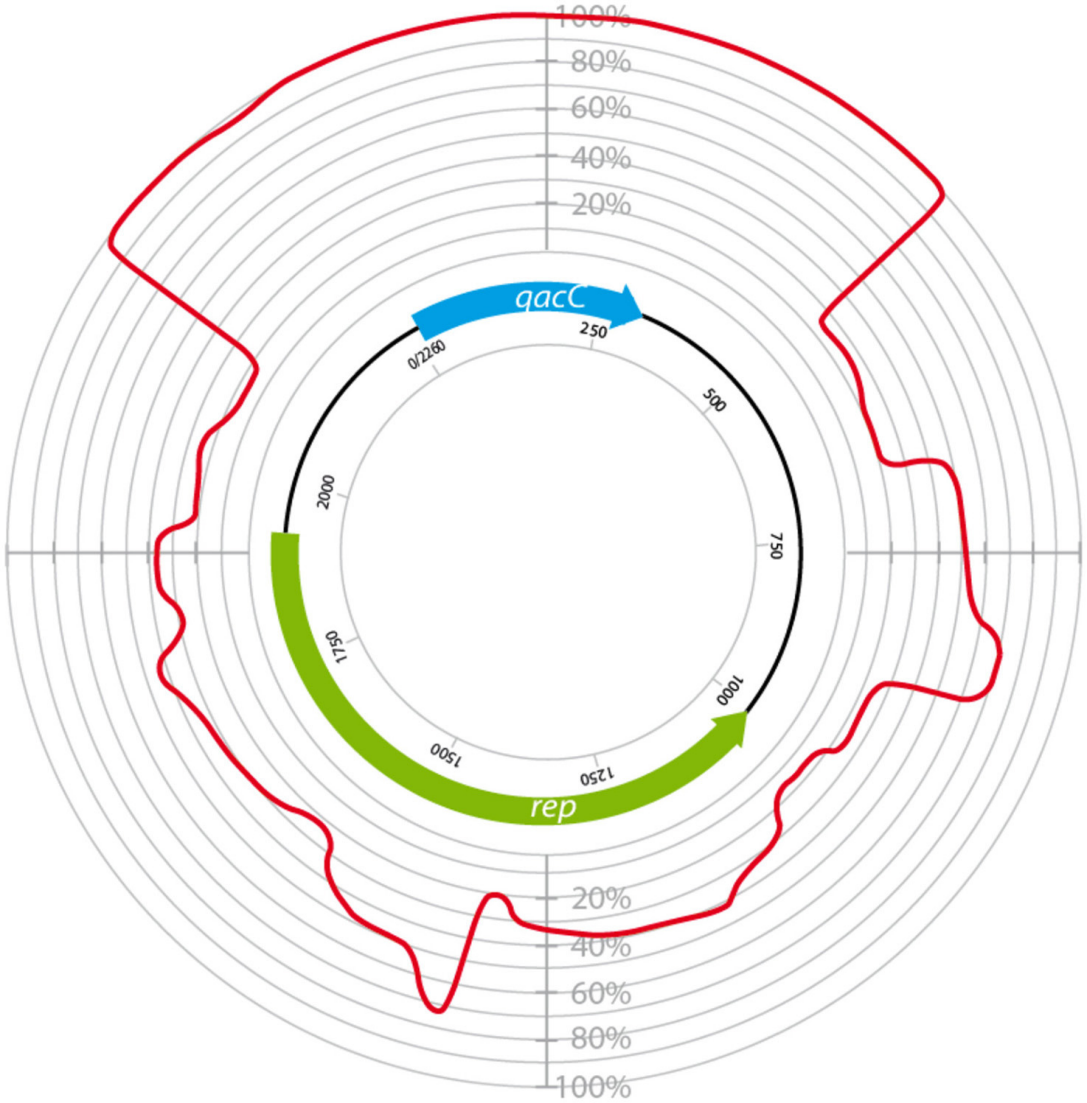
**Circular homology plot based on the nucleotide alignment between QacC plasmids**. The figure is based on an alignment after gap removal of pNVH99, pWBG754, pSA1308, pLUH01, pKH8, pSH4126, pPI-2, pSW49, pSK89, pWBG32, pSepCH, and pSM52, which were reopened for alignment at the start of *qacC*. The indicated length of *rep* is the average of the analyzed genes.

Surprisingly, the conservation of *qacC* at the nucleotide (nt) level is also nearly absolute: in addition to the two mutations resulting in the aa substitutions, only one synonymous mutation has been encountered so far (an A → G mutation at nt position 81 in pNVH99 of *S. aureus*). Due to this strict conservation, which is indicative of a recent bottle-neck selection event, phylogenetic analysis of the QacC protein or its gene is not possible, whereas inclusion of the sequences flanking *qacC* introduces informative variation. Analysis of a number of complete *qacC*-bearing RC-plasmid sequences (selected for their length of approximately 3 kb) reveals that these are variable, apart from their strictly conserved *qacC* gene plus its flanks, as shown in Figure 1.

The plasmids used in Figure 1 fall into two types depending on their *rep* gene, which we define as Type I and Type II, respectively, as summarized in Table 1. Rep1, the determinant for Type I plasmids, is typically 282–307 aa long while Rep2, indicative of Type II, is 332–334 aa. Rep2 homologs were previously described as RepNVH99 (Bjorland et al., 2001) and REP416 (Alam et al., 2003). Both types of plasmids mostly lack other recognized genes and their *rep* is found invariably upstream of *qacC* on the opposite strand, so that the two genes are divergently transcribed.

**Table 1:** **Plasmids containing QacC used in the analysis**.

**Type**	**Characteristic**	**Plasmid name**	**Accession number**	**Plasmid length (bp)**	**Isolated from**	**Remarks**
**Short RC-plasmids**	**Examples**						
Type I	QacC and Rep1	pSepCH	AY092027	2391	*S. epidermidis*	
		pSK89	M37889	2390	*S. aureus*	
		pWBG32	M33479	2389	*S. aureus*	Incomplete plasmid sequence, Rep1 not annotated
Type Ia	QacC', QacC, and Rep1	pPI-2	AB125342	2779	*S. warneri*	Extra G 137 bp downstream of QacC
		pSM52	JX898993	2782	*S. aureus*	Extra G 137 bp ds downstream QacC
		pSW49	AM040730	3552	*S. warneri*	A9S mutation. Extra G 137 bp downstream of QacC, contains Rep2 fragment
		pSW174	AM040729	5767	*S. warneri*	A9S mutation. Extra G 137 bp downstream of QacC, also contains REP2
Type II	QacC and Rep2	pSA1308	AB254848	2756	*S. aureus*	Extra G 137 bp downstream of QacC
		pWBG754	GQ900396	2241	*S. aureus*	
		pNVH99	AJ296103	2239	*S. aureus*	Silent mutation in QacC
		pLUH01	FR714928	2241	*S. aureus*	Extra G 137 bp downstream of QacC. QacC not annotated
		“pSH416”	AY121857	2425	*S. aureus*	Unnamed plasmid from strain SH416. Incomplete plasmid seq. Extra G 137 bp downstream of QacC
Type IIa	QacC', QacC, and Rep2	pSK108	GQ900464	2418	*S. epidermidis*	Extra G 137 bp downstream of QacC
		pKH8	U50077	2417	*S. aureus*	Extra G 137 bp downstream of QacC
Type III	Direct repeats flank QacC	pST827	Z37964	2831	*Staphylococcus* sp.	Isolate described as “meat-associated.” Contains Rep2
		pSP187	AM040731	5550	*S. pasteuri*	Contains a different Rep gene plus other genes
**Long conjugative plasmids**		**Examples**				
Type III	Direct repeats flank QacC	pSAP016A	GQ900381	43,807	*S. epidermidis*	
		pSAP082A	GQ900434	44,116	*S. aureus*	
		pGO1	FM207042	54,000	*S. aureus*	
		pSK41	AF051917	46,445	*S. aureus*	
		pLW043	AE017171	57,889	*S. aureus*	
		pSAP079A	GQ900432	47,322	*S. aureus*	Contains 3 direct repeat units at start of upstream DR1 unit
		pSAP069A	GQ900422	42,198	*S. aureus*	DR3 unit downstream of QacC is truncated
**Unclassified**						
	QacC	“pSH651”	AY121858	1545	*S. aureus*	Unnamed plasmid from strain SH651. IS431 directly downstream of QacC. Incomplete plasmid sequence

Excluded are QacC sequences from GenBank entries that contain insufficient flanking sequences, double entries, or contigs from genomic sequences that were not (yet) fully assembled.

Even within their types, Rep1 and Rep2 are still less conserved than QacC: Rep1 is conserved for 77% at the aa level (68% at nt level), and for Rep2 these percentages are 82 and 78%, respectively. The difference in nt conservation between *rep* and *qacC* dictates these genes did not co-evolve on the RC-plasmids where they now reside. Instead, the observation can only be explained by assuming that *qacC* was recently incorporated into plasmids with variable *rep* genes, an event that occurred so recently that third-base variation has not yet been introduced.

**Figure 2:**
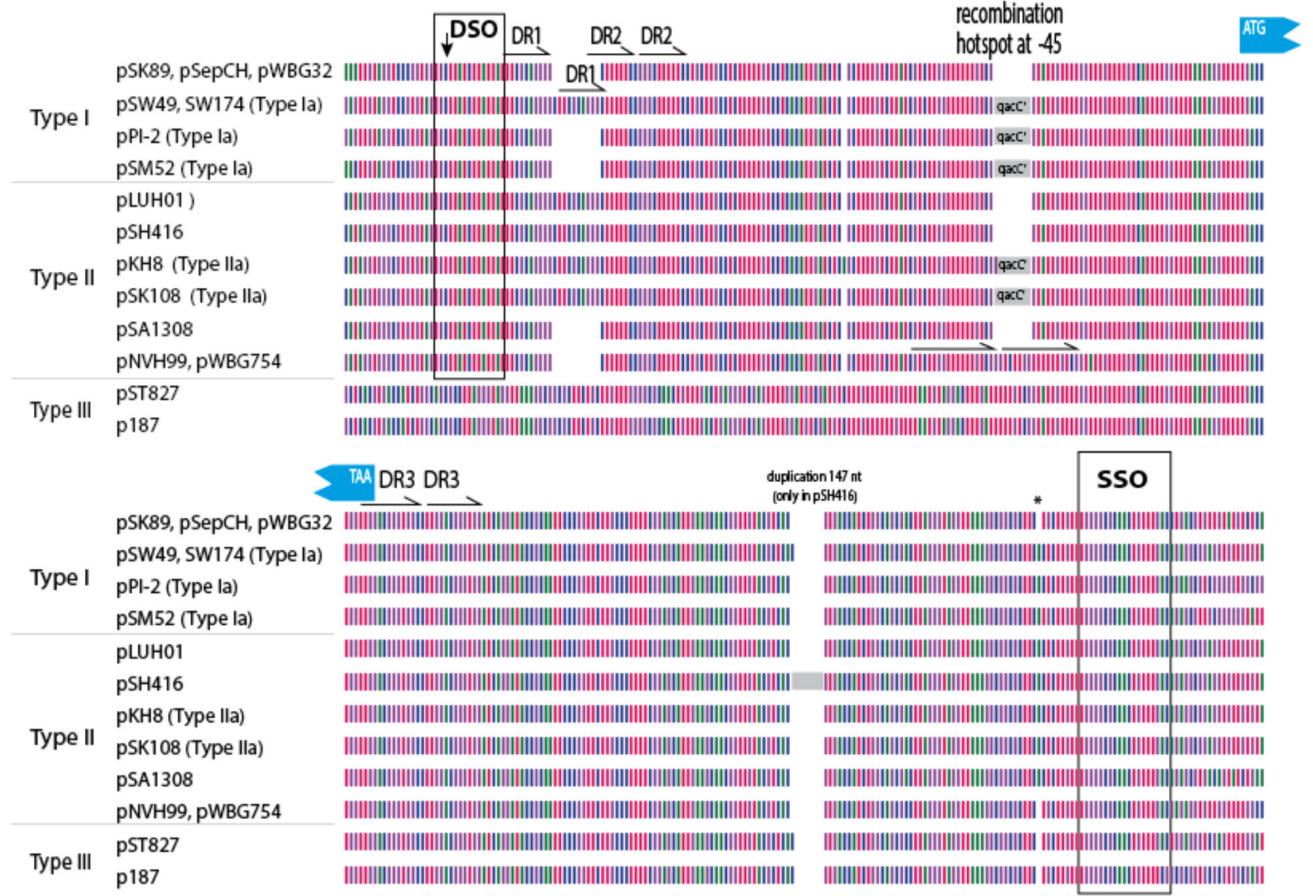
**Graphical representation of 200 ***qacC*** upstream (top) and downstream (bottom) sequences**. Nucleotides are represented by color with A (red), T (purple), G (green), and C (blue). DSO, double-strand origin of replication; DR, direct repeat; SSO, single-strand origin of replication. Other features indicated are discussed in the text.

In an attempt to explain the finding of a conserved *qacC* in these variable backgrounds we compared the flanking sequences of *qacC* in the RC-plasmids listed in Table 1. Key features of the multiple DNA alignment are summarized in Figure 2.

Upstream of *qacC* 162 nucleotides are identical in Type I and Type II plasmids except for two regions: a variable number of direct repeats (DR1) is present toward the distal end of the upstream flank, and an 8 nt repeat insert is found in two Type II plasmids, in a region where a *qacC'* insert is found in a number of other plasmids, which will be revisited in the next section. The distal end of this 162-long upstream region starts with the DSO, the nature of which will be discussed at the end of this paper. Beyond the DSO, moving further away from *qacC* in the upstream direction (toward the start of the replication gene), the sequences diverge between Types I and II (Figure 2). Downstream of *qacC*, Type I and Type II plasmids share 164 nt before their sequences diverge. The exception is pSH416, which contains a large insert, although it cannot be excluded that this is the result of an assembly error.

Thus, the borders of a conserved *qacC* locus shared between Types I and II plasmids is defined from 162 nt upstream (bearing a DSO element) to 164 nt downstream of the *qacC* open reading frame, and includes an SSO element. We proposed this “*qacC* element” has recently been spread over short RC-plasmids.

### Plasmids with a truncated *qacC'* in front of a functional *qacC*

As has been previously described, a number of short RC-plasmids contain a repeat of 142 nt, composed of the first 97 coding nt of *qacC*, together with 45 upstream nt (indicated by a gray box in Figure 2). The fragmented open reading frame is frequently annotated as *qacC'* (previously called Delta-*qacC*) but the gene fragment is too short to produce a functionally active protein (Leelaporn et al., 1995). Phenotypic evidence from pSM52 (Costa et al., 2013) or pSW49 (Bjorland et al., 2006) suggests that the promoter of *qacC* is present on the 45 nucleotides that separate the *qacC'* fragment from the active, complete gene (the promoter is also present in front of *qacC'*).

**Figure 3:**
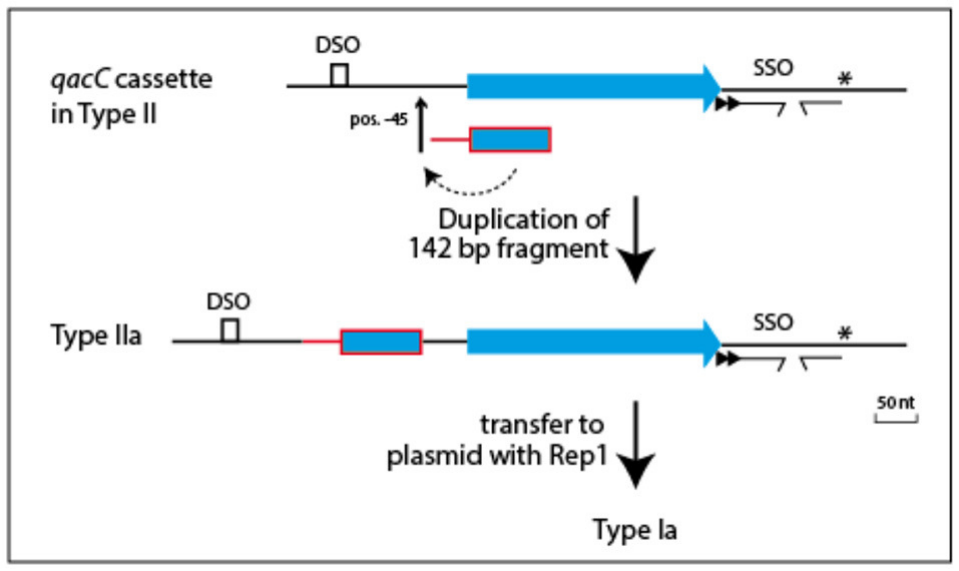
**Model for formation of the ***qacC'*** fragment present in Types Ia and IIa plasmids**. A single nt insertion 137 nt downstream of *qacC* (indicated by the asterisk) is present in some Type II plasmids (e.g., pLUH01) and conserved in both Types Ia and IIa. This is evidence that the duplication of the 142 nt-long *qacC'* fragment, inserted at position −45, occurred in a Type II plasmid to form Type IIa, after which the complete locus was mobilized to give Type Ia.

Plasmids containing the 142-nt duplication of *qacC'* are designated Type Ia or Type IIa, depending on their Rep (Table 1). That this partial duplication can be variably present on both types can be explained in two ways. Either the *qacC* locus was distributed to Type I and Type II plasmids first, after which an identical *qacC'* duplication occurred independently in both, or the duplication occurred first and the locus with or without the duplication spread independently in both types of plasmids. Analysis of the downstream sequences provides a clue as to which scenario is more likely. A single-nucleotide insertion is found 137 nt downstream of *qacC* in all known Type Ia and Type IIa loci (indicated by an asterisk in Figure 2), and it is also present in some Type II loci (e.g., pSA1308 or pLUH01, see Table 1). It is therefore proposed that the duplication occurred in such a Type II plasmid (Figure 3), after which the whole locus including the duplication was distributed over Type I plasmids. This observation supports the hypothesis that the QacC locus is transferable.

A few Type Ia plasmids contain multiple *rep* (pseudo)genes, for example pSW49 of *S. warneri* (Bjorland et al., 2006). This plasmid of 3552 bp length contains, besides *qacC', qacC*, and *rep1*, a truncated *rep2* pseudogene. Although occasionally RC-plasmids are encountered that contain two different but active replication proteins (e.g., pMRI5.2 of *Lactobacillus plantarum*, Cho et al., 2013), in most cases only one functional replication initiation protein is present. Plasmid pSW49 is possibly a fusion product of a Type Ia and a Type IIa plasmid, in which only one copy of the *qacC'-qacC* locus was maintained, while Rep2 was truncated. Likewise, *S. warneri* plasmid pSW174 is likely a fusion plasmid that in this case retained both Rep genes.

Alam et al. (2003) defined a “Type 3” class based on the plasmid of *S. aureus* strain SH651. This unnamed and incompletely sequenced plasmid (called pSH651 in Table 1) contains an insertion sequence directly downstream of *qacC*. It further contains a 122 nt direct repeat upstream of *qacC*. The total length of this plasmid is not recorded and further sequences have not been made available, so the exact nature of this plasmid cannot be determined at this stage. However, the 122 nt repeat is part of a sequence that is also repeated in other RC-plasmids bearing *qacC*, which we call Type III.

### Type III *qacC* plasmids, progeny or precursor?

The prototype of Type III plasmids is pST827 (Table 1), sequenced from a strain of unspecified “*Staphylococcus* sp.” isolated from food (Heir et al., 1995) (an incorrectly assembled version of this plasmid appeared in Alam et al., 2003). In Type III plasmids, *qacC* is flanked by two identical 229 nt-long sequences so that the upstream and downstream flanks of *qacC* are identical, except for one mismatch (an extra T present in the downstream unit). For this reason, Type III plasmids do not match the conservation of the upstream *qacC* flank (Figure 2) and were excluded from the homology plot in Figure 1.

**Figure 4:**
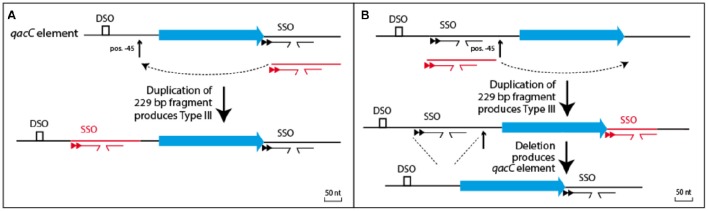
**Relationship between Types I/II and Type III plasmids**. **(A)** Type III could be formed from the *qacC* element by a single duplication event whereby the downstream flank is inserted into a hotspot for recombination, positioned 45 nt upstream of *qacC*. **(B)** Alternatively, a precursor can be hypothesized with *qacC* next to DSO and SSO. A duplication of the SSO-bearing segment would result in Type III, after which a deletion would produce the *qacC* element.

Two models can be proposed how the *qac* locus of Type III plasmids relates to Types I and II, shown in Figure 4. The simplest model (Figure 4A) proposes that Type III is formed from a Type I or II *qacC* plasmid by duplication of the downstream flank inserted into the upstream flank, at position −45 nt. Alternatively (Figure 4B), Types I and II are formed from Type III, which requires a hypothesized precursor in which upstream sequences have been duplicated into the downstream flank. Although that model requires two steps, it may be the more likely scenario, because in the hypothesized precursor a DSO and SSO would be separated by 316 nt, a distance that is more typical for staphylococcal RC-plasmids that only contain a *rep* gene (e.g., pSK6 of *S. aureus*, acc. nr. NC_001995).

Table 1 lists pSP187 from *Staphylococcus pasteuri* as a second example of a Type III RC-plasmid. It contains a different replication gene as well as a few other genes—it may have resulted from a recombination with another plasmid. So far, pST827 and pSP187 are the only completely sequenced short RC-plasmids containing a Type III *qacC*.

The *qac* locus of Type III RC-plasmids can also be found on long conjugative plasmids; seven of these (Table 1) were used for comparative analysis (a graphical representation is given in Figure S1). In these plasmids two transposase genes that are part of Insertion Sequence IS257 copies border the locus. In between these ISs a *rep1* gene is present. This suggests that a complete Type I plasmid was incorporated into a conjugative plasmid, after which recombination events duplicated the SSO-bearing fragment and deleted the DSO flank immobilized the plasmid. The public database does not contain short *qacC* RC-plasmids of Type III that contain rep1.

### A hotspot of recombination upstream of *qacC*

We noted that the insertion of the duplication observed in Type III plasmids occurred at the same position as the insertion of *qacC'* in Types Ia/IIa. This suggests the −45 position is frequently subject to recombination. Indeed, in two Type II plasmids (pWBG754 and pNVH99), 18 nt are repeated here, replacing 10 nt of original sequence, thus resulting in an insertion of 8 nt in the alignment (Figure 2). That the position 45 nt upstream of *qacC* is a frequent target for recombination may be due to the local physical properties of the sequence. An analysis of the DNA structural properties revealed this is an AT-rich region that is curved and will easily melt.

### Other Qac members of the SMR family

Relatively few sequences are available for Qac members of the SMR family other than QacC. Less than 10 records each are currently available for QacG, QacJ, or QacH, and a number of these are contigs from genome or metagenomic sequencing projects. All these plasmids contain a *rep2* homolog.

The nucleotide sequences of five QacG plasmids (four RC-plasmids and one segment of a conjugative plasmid) listed in Table 2 produce a similar conservation pattern as observed for QacC plasmids: the open reading frame of *qacG* is 99% conserved (5 mismatches only) whereas *rep2* is only 76% conserved. Again, the nearly complete conservation of *qacG* is extended to its flanking sequences, encompassing the DSO and SSO, although the homology is continued 22 nt upstream of the DSO nick site. In contrast, the five analyzed QacJ sequences (three from contigs) are stronger conserved for *rep2* (91%) than for *qacJ* (83%) and the homology of the rest of the plasmids suggests they are closely related (data not shown). These observations show that there is no functional selection that would prevent SMR-type *qac* genes from accumulating nt mutations: *qacG* seems to have been around long enough to collect a limited number of mutations already, in contrast to *qacC*. There are not enough completely sequenced *qacH* plasmids publically available to assess their homology.

**Table 2:** **Plasmids containing QacG, QacJ, or QacH used in the analysis**.

**Gene (protein accession nr)**	**Plasmid name**	**Accession number**	**Plasmid length (bp)**	**Isolated from**	**Remarks**
QacG (O87866)	pST94	Y16944	2267	*S. aureus*	
QacG (YP_006938120)	pSA052A	NC_013332	32,445	*S. aureus*	Conjugative plasmid, contains truncated Rep2
QacG (WP_002477903)	“BVSO” from strain BVS058A4	AGZV01000050	2263	*S. epidermidis*	S32T. Rep is annotated as “hypothetical” and a probable assembly error (duplication) at the circularization border was corrected
QacG (ALS73700)	pRIVM1076	CP_013624	2216	*S. aureus*	
QacG (WP_015740450)	pW33578	NZ_kk038130	2538	*S. aureus*	Contig
QacJ (EQM91153)	pS1d	AUPS01000033	2649	*S. aureus*	Contig
QacJ (CAD55144)	pNVH01	NC_004562	2650	*S. aureus*	
QacJ (AAB47993)	pKH4	U81980	2487	*S. aureus*	
QacJ (WP_052996450)	pNA32	NZ_CTWR01000064	2639	*S. aureus*	Contig
QacJ (WP_052996449)	p92271	NZ_CUFY01000074	2613	*S. haemolyticu*	Contig
QacH (CAA76544)	pST2H6	Y16945	2378	*S. saprophyticus*	Incomplete plasmid sequence
QacH (AAO83011)	pTEF1	AE016833	66,320	*E. faecalis*	Not included in the analysis
QacH (ACC78797)	–	EU622634	225	*S. haemolyticus*	Partial open reading frame only

**Figure 5:**
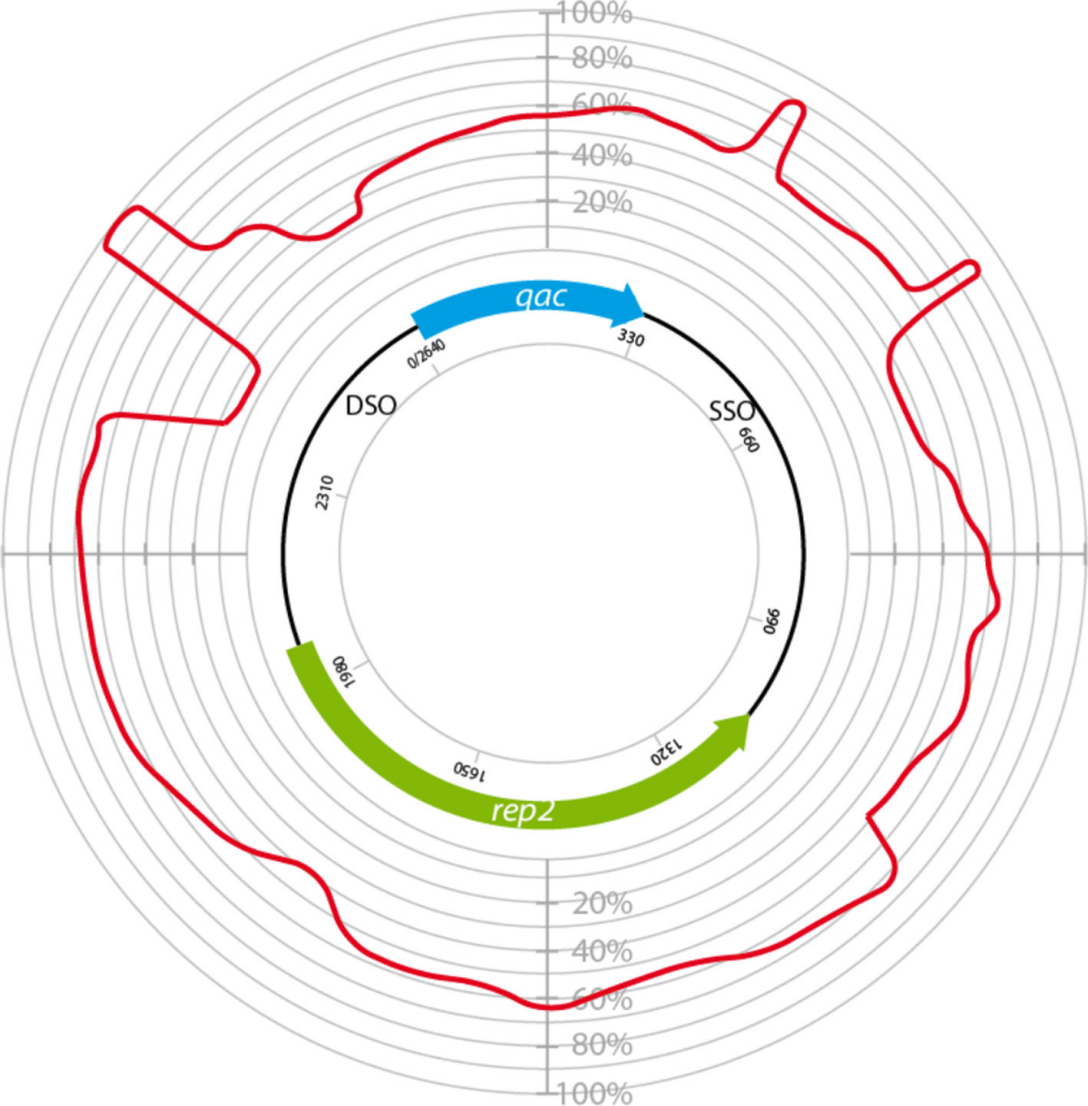
**Circular homology plot of plasmids bearing variable ***qac*** genes**. The homology is based on 4 QacC plasmids (Type II, Table 1) and 5 QacG, 5 QacJ, and 1 QacH plasmids, listed in Table 2.

The homology between plasmids bearing *qacC* (Type II), *qacG, qacJ*, and *qacH* was used to identify conserved regions (Figure 5). These were restricted to the DSO, a short region immediately downstream of *qac*, and the distal end of the SSO region.

### Conserved DSO and SSO in three classes of *qac* genes

**Figure 6:**
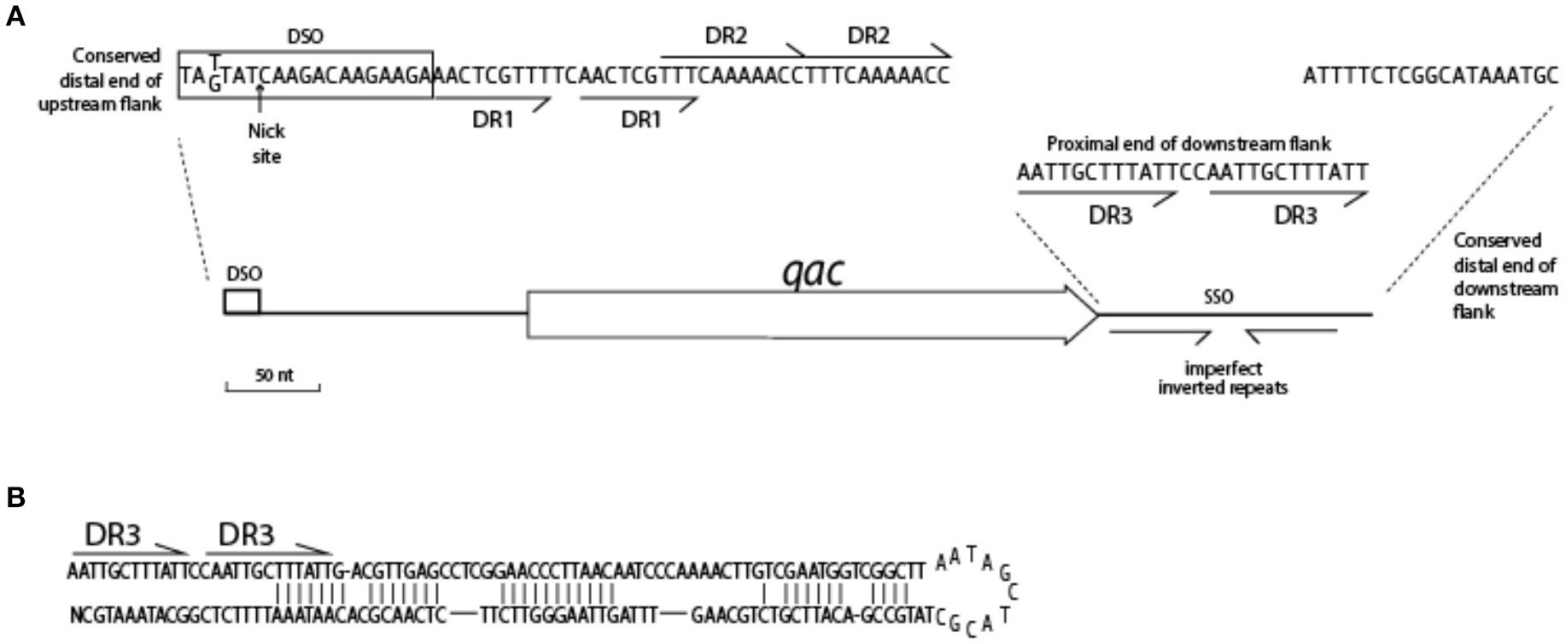
**Conserved features of the Qac locus**. **(A)** Sequences indicated are highly conserved in the flanks of 25 *qacC, qacG*, and *qacJ* genes. **(B)** Hairpin structure of SSO for QacC plasmids.

Adding sequences of Type I QacC plasmids to the alignment on which Figure 5 was based identified conserved sequences essential for replication, summarized in Figure 6. The distal end of the 165 nt-long QacC upstream flank including the DSO is strictly conserved, though the third base is G instead of T in Type I QacC plasmids only. The direct repeat unit DR1 of QacC plasmids is also present in all QacG plasmids, two QacJ plasmids and in the single QacH plasmid that was analyzed. Besides in QacC plasmids, the DR2 unit is repeated in two QacJ plasmids (the three other QacJ sequences contain an alternative DR2). In all plasmids, the DSO is followed by a stretch of low complexity sequences containing multiple short homo-nucleotide repeats. The rest of the upstream flank is not conserved between these Qac plasmids.

The downstream flank of all *qac* genes is more strongly conserved than the upstream flank. It starts with a 12 bp-long direct repeat, (AATTGCTTTATT, indicated as DR3 in Figure 6), which is completely conserved in case of *qacC* and *qacJ*; the first unit is mutated in *qacG* and both units contain mutations in *qacH* sequences. The very distal end of the downstream flank contains 18 nt that are strongly conserved (*qacJ* contains one mismatch in the last T). Beyond this, homology is lost. Weakly conserved inverted repeats are present in the downstream flank, which can form a hairpin structure (Figure 6B) related to the function of SSO.

The conserved sequence of the DSO given in the box of Figure 6 is the strand on which the *qac* gene is found. The arrow indicates the position where Rep introduces a nick in the complementary strand, which initiates replication of the plus strand (Gruss et al., 1987; Khan, 2005; Bikard et al., 2010). RC-plasmids are divided into different families depending on the type of replication protein and the DSO sequence this protein recognizes. Originally, four eubacterial RC-plasmid families were recognized (del Solar et al., 1998) which were extended to over 12 (Khan, 2005) and now 17 (Ruiz-Masó et al., 2015) plasmid families. Based on aa similarity, Rep1 of Type I plasmids belongs to the pC194-family (Seery et al., 1993), a representative of the *incB* incompatibility group (Thomas et al., 1990). The conserved DSO resembles that of pC194 with only one mismatch and one insertion (the DSO of pC194 is TATTAT↓CAAGATAAGAAAGA, Bikard et al., 2010). The nature of Rep2 is less clear, though it is more closely related to Rep of pC194 than to Rep proteins of the other families; information on its *inc* group could not be identified. Rep2 recognizes the same DSO as Rep1 with the exception of the polymeric nucleotide positioned four bases upstream of the nick site, which is G instead of T.

In a number of well-studied RC-plasmids, the nick site is positioned in the loop of a stem-loop structure that is formed by inverted (palindromic) repeats (Ruiz-Masó et al., 2015). However, from the DSO and sequences surrounding the nick site in Qac plasmids inverted repeats are absent. Lack of hairpin structures in a DSO has been observed before in other members of the pC194-family, for instance in pUB110 of *Bacillus subtilis* (Alonso et al., 1988). A functional DSO further depends on a binding element for the Rep dimer. This binding element is usually less conserved than the nick site and in case of the pC194 family typically contains inverted repeats (del Solar et al., 1998; Bikard et al., 2010). Since these are absent in case of Qac, we propose that two sets of direct repeats following the nick site function as the binding region for Rep1 and Rep2.

The conserved SSO found in the downstream flank of *qacC* belongs to the *ssoA*-type (Kramer et al., 1998). Irrespective of whether Qac is present or absent, this particular SSO sequence is almost exclusively found in *Staphylococcus* plasmids, in accordance to experimental evidence that SSO sequences are host-specific (Kramer et al., 1998), although promiscuous within the genus.

In the Type III QacC plasmid pST187, where the SSO flank is duplicated, the event has replaced the DSO. Lack of a recognizable DSO element has been pointed out before (Bjorland et al., 2007). These authors proposed an alternative DSO element, and in combination with its unique Rep gene it was suggested that pSP187 belongs to RRC-group VI plasmids (Bjorland et al., 2007).

### A model of Qac mobility

**Figure 7:**
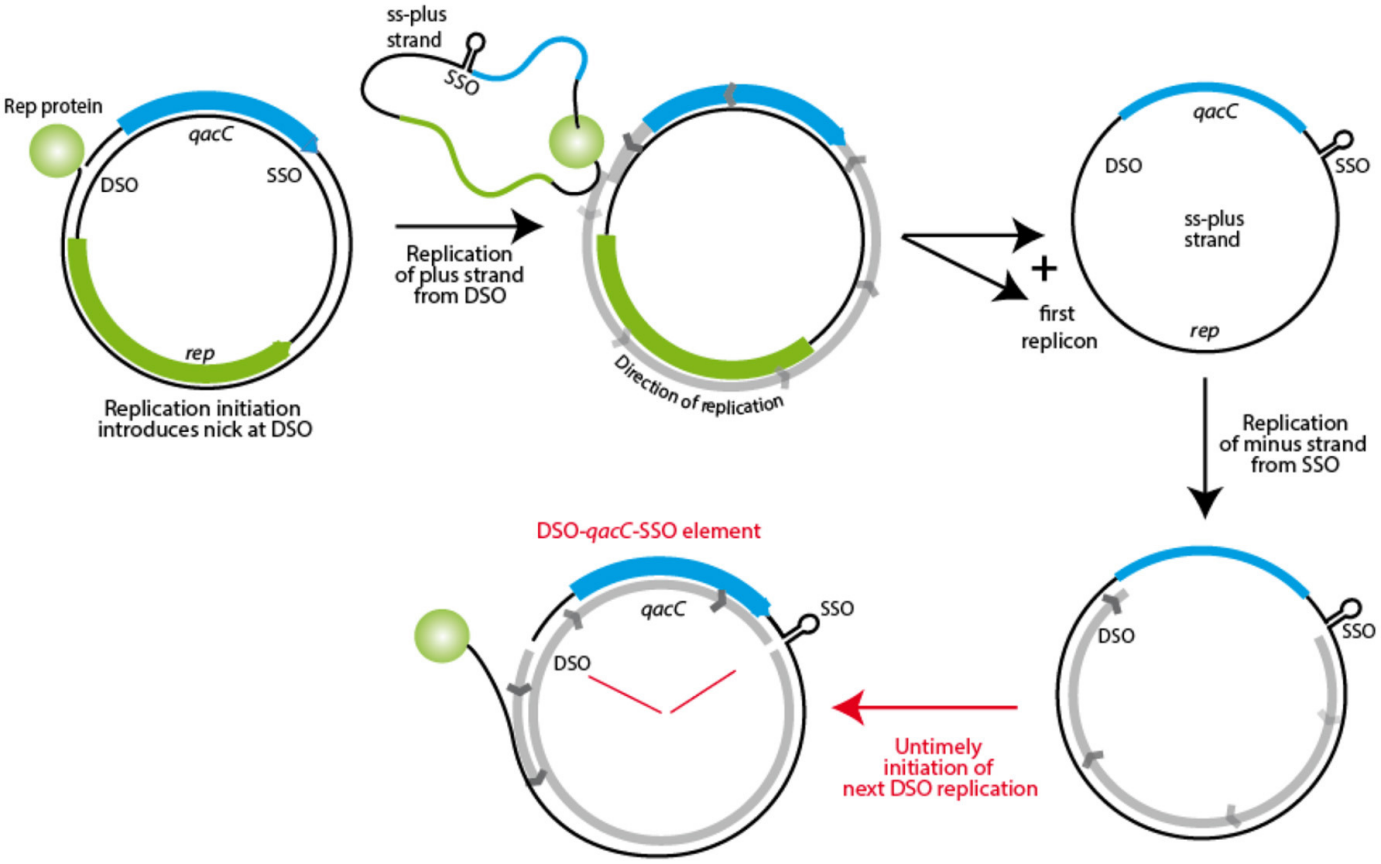
**Model for mobility of the ***qacC*** locus**. The first step in RC-replication is production of the plus strand, resulting in the template plus strand as an ssDNA intermediate. This is subsequently replicated from the SSO, but when a new nick is introduced in this DNA before replication is completed, a fragment containing *qacC*, flanked by nicked DSO and SSO is the result. This DSO-*qacC*-SSO element could then be incorporated into an alternative plasmid.

The fact that Qac is positioned in between SSO and DSO on RC-plasmids may explain how it is mobilized. During rolling circle replication, the plus strand is formed from the nick site, with the replication fork moving all the way round toward the SSO, to continue beyond the original nick site. As Khan describes: “Once the replication fork reaches the termination site, i.e., the regenerated DSO, DNA synthesis proceeds to approximately 10 nucleotides beyond the nick site. (…) The SSO *[is] generally located immediately upstream of the* DSO such that [it is] not exposed in a single-strand form until the leading strand has been almost fully synthesized” (Khan, 2005). Initiation of replication is tightly regulated, and how re-initiation is inhibited until the cycle is completed is well understood for some RC-plasmid families (e.g., pT181, reviewed in Ruiz-Masó et al., 2015), but for the pC194 family, to which Qac RC-plasmids belong, the regulation is not clear (Ruiz-Masó et al., 2015). The presence of a gene (*qacC*) in between SSO and DSO is an anomaly for these plasmids, as the *sso* is usually located close to the *dso* (Ruiz-Masó et al., 2015). During replication, which starts from DSO (with *qacC* being replicated last) an intermediate stage exists when the single-strand positive strand is covalently bound to Rep at the nick site, to be recircularized when Rep is released. This circular ssDNA is completed to dsDNA starting at the SSO hairpin, again with *qacC* being replicated last, as shown in Figure 7. If, however, a new nick is introduced in DSO before the minus strand replication round is completely finished, a nicked DNA fragment spanning from the nick site to the SSO, with *qacC* in between, would be formed. This could be incorporated in an acceptor plasmid by homologous recombination, making use of host recombination proteins. Untimely initiation of replication would normally be prevented when SSO and DSO are close together, but it is quite possible that the presence of a *qac* gene in between these elements upsets the timing of events.

### QacC is not the only transferable gene employing SSO and DSO sequences

In search of support for our model, we looked for other genes located between SSO and DSO of RC-plasmids. In species other than *Staphylococcus* this is quite common for *rep* or *mob* genes, and a variety of other genes can be found to separate the two replication elements (e.g., Alonso et al., 1988; Andrup et al., 2003; Alegre et al., 2005; Wu et al., 2007; Zhang et al., 2010, 2011). None of these genes in RC-plasmids from genera other than *Staphylococcus* share significantly higher homology compared to the rest of their plasmid background, thus there is no indication of gene transfer. Whether other genes on RC-plasmids from genera other than *Staphylococcus* use this mode of mobilization is currently not known.

However, a second example exists of a gene likely to be transferred by the same mechanism. The gene *lnuA*, resulting in lincosamide resistance, is located in between SSO and DSO on RC-plasmids of *Staphylococcus* species, and again it is stronger conserved at the nt level than the sequences outside the proposed mobile locus. As was observed for *qacC*, the gene can be present on long conjugative plasmids as well as RC-plasmids. In the latter case, the only other gene usually present is *rep2*. It has been observed before that *lnuA* in such plasmids is more strongly conserved than *rep* (Lüthje et al., 2007). Comparison of nine RC-plasmids by multiple nt alignment identified the segment containing DSO, *lnuA* and SSO (694 bp) as 92.7% conserved, compared to 77% for the *rep* gene (coding sequences only) or 74.4% for the plasmid excluding the DSO-*lnuA*-SSO segment (ignoring a weakly conserved region). The conserved flank upstream of *lnuA* is 78 nt long and the downstream flank is for 160 nt conserved, though the number of direct repeats immediately downstream of *lnuA* varies. The circular homology plot is available as Figure S2.

## Discussion

The data presented here suggest a recent spread of *qacC* within and between Type I and Type II RC-plasmids. For the DSO-*qacC*-SSO element to end up in a new plasmid, an acceptor plasmid would probably have to be present together with the donor plasmid in one cell, or at least within the same bacterial population. This requirement has to obey plasmid incompatibility rules. Possibly, Type II, Rep2-containing plasmids are compatible with Type I plasmids that contain Rep1, an assumption that can be experimentally tested. However, our observations further identified spread of the element between plasmids with the same Rep gene. It has to be assumed that in such a case the transfer required cell-cell contact between different plasmid-bearing bacteria, the details of which have not been investigated.

It may not be a coincidence that both examples of a mobile DSO-gene-SSO element identified contain an antimicrobial resistance gene. Possibly, the recent spread of *qacC* or *lnuA* between *Staphylococcus* plasmids and strains has been selected for by use of disinfectants and antibiotics, and the availability of multiple *Staphylococcus* plasmid sequences have enabled this discovery. The accumulation of a few mutations suggests the spread of *qacG* is less recent than that of *qacC*.

Of the other *qac* genes, *qacG* but not *qacJ* is likely to have been mobilized recently, based on its pattern of conservation. It is not clear why evidence is lacking that *qacJ* has recently spread; possibly, the selection for this gene is weaker or the population bearing *qacJ* has not recently passed a bottleneck. QacJ plasmids were first described from equine isolates of *S. aureus, Staphylococcus simulans* and *Staphylococcus intermedius* (Bjorland et al., 2003), though human *S. aureus* (pS1d, pNA32) and *Staphylococcus haemolyticus* (p92271) isolates can also contain QacJ plasmids. Currently there are too few QacJ plasmid sequences available to draw conclusions about its possible mobility.

Although no experimental evidence has been obtained to support our model of gene mobility, for which a location between the DSO and SSO is crucial, we make a strong case for recent spread of *qacC* between various *Staphylococcus* RC-plasmids, based on its nearly complete conservation in variable plasmid backgrounds. The hypothesis can be tested by use of a constructed larger sized Type I plasmid with a resistance gene A located between SSO and DSO, and a smaller compatible Type II plasmid with an alternative resistance gene B located at the same position. After double transformation of both plasmids into *S. aureus* under double selection, plasmid DNA could be isolated from the population and physically separated by size. By retransformation of the large plasmid population under selection for resistance gene B, a minor population of recombined plasmid DNA might be identified. It is expected that a transfer of gene B does not occur at the same rate in case it is not located in between DSO and SSO.

The discovery of an independent second gene, *lnuA*, whose higher level of conservation between different plasmids compared to *rep* is again bordered by DSO and SSO, strengthens the proposed model. This is the first evidence of mobile genes that can be transferred between plasmids without the aid of insertion sequences or transposases. We propose to call this type of mobile DNA an “DSO-gene-SSO” element.

## Author contributions

TW collected the plasmid sequences and performed the alignments. DU provided analysis tools for the initial screens and advised on the presentation of data. HI assisted in the selection of data to be shown and in data presentation and description. All authors contributed to writing of the manuscript.

### Conflict of interest statement

The authors declare that the research was conducted in the absence of any commercial or financial relationships that could be construed as a potential conflict of interest.
