# Supplementary material for: The qacC Gene Has Recently Spread between Rolling Circle Plasmids of Staphylococcus, Indicative of a Novel Gene Transfer Mechanism
Source: Front Microbiol. 2016 Sep 27;7:1528. doi: 10.3389/fmicb.2016.01528 (PMC5037232; doi:10.3389/fmicb.2016.01528)
Supplement: Supplementary file 1 [file DataSheet1.DOCX]

**
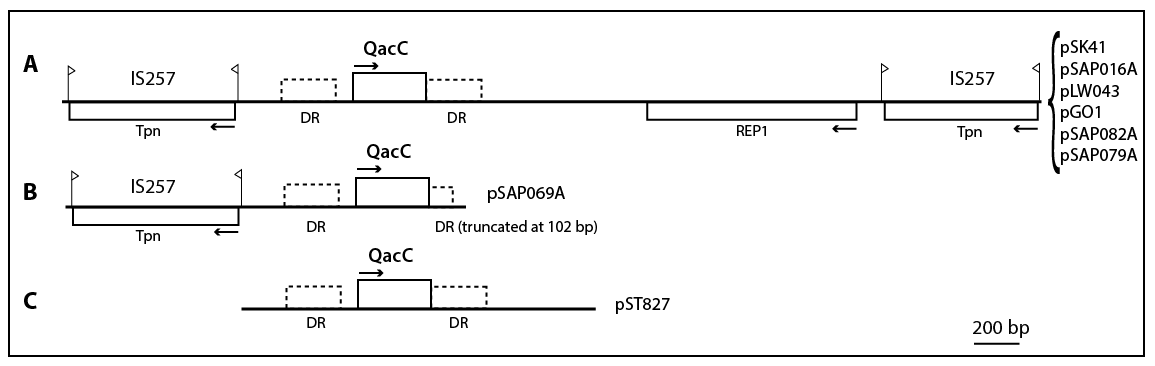
**

**Figure S1**. The Type III QacC locus in conjugative and RC-plasmids.

Panel A: QacC and surrounding genes found in 6 long, conjugative plasmids (listed to the right). The 229-nt direct repeat (DR) is indicated as dotted boxes. Arrows indicate the direction of gene transcription. Tpn is a transposase and flags indicate the border of IS257.

Panel B: the truncated Type III locus in conjugative plasmid pSAP069A.

Panel C: Type III locus in RC-plasmid pST827. Solid black lines indicate homology between the three panels.

**
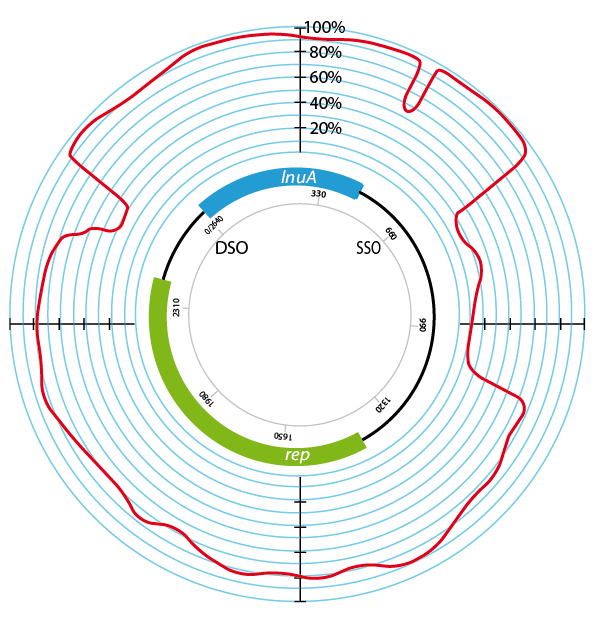
**

**Figure S2. Circular homology plot based on 9 LnuA RC-plasmids.**

The homology is based on 9 RC-plasmids containing *lnuA*: pLNU2, pLNU8, pLNU3, pLNU4, pUR5425, pLNU7, pLNU6, pLNU5 and pKH21.
